# Antithrombotic effects of Huanglian Jiedu decoction in a rat model of ischaemia-reperfusion-induced cerebral stroke

**DOI:** 10.1080/13880209.2021.1942505

**Published:** 2021-07-01

**Authors:** Huan Liu, Xiaoyan Chen, Yanling Liu, Chunjuan Fang, Shaofen Chen

**Affiliations:** Jiangxi University of Technology, Nanchang, Jiangxi, China

**Keywords:** Chinese medicines, thrombosis, platelet aggregation, baicalin, berberine, geniposide

## Abstract

**Context:**

Huanglian Jiedu Decoction (HLJJD) has a variety of pharmacological activities, such as anti-inflammatory and neuroprotection against ischaemic brain injury.

**Objectives:**

This *ex vivo* study explores its antithrombosis activity and inhibition of platelet aggregation.

**Material and methods:**

To study the antithrombosis activity of HLJJD *ex vivo*, saline, or HLJDD (100, 200, and 500 mg/kg) was treated prophylactically by gavage for 3 days in Wistar rats (*n* = 4). Based on the rat model of transient middle cerebral artery infarction (MCAO) or normal rats (*n* = 4), the antithrombotic activity in the normal group and HLJDD subgroups on prothrombin time, thrombus weight, platelet aggregation, and others was evaluated, followed by the antiplatelet aggregation of its main components (*n* = 4).

**Results:**

The weight of the thrombus increased significantly at 24 h after MCAO onset. HLJJD did not influence the change of PT, but significantly inhibited thrombosis by 12.5, 20.0, and 20.5% in reducing the dry weight of thrombus, and blocked collagen-induced platelet aggregation by 25.5, 39.0, and 42.7% and adhesion of blood platelet by 17.3, 26.2, and 27.3%. The IC_50_ value of HLJJD on collagen-induced platelet aggregation was 670 mg/kg. Geniposide only facilitated antiplatelet aggregation induced by collagen, but not AA or ADP. Both baicalin and berberine showed markedly antiplatelet aggregation induced by all activators. The antithrombotic activity of baicalin was relatively higher than that of berberine (35.0–47.8% *vs.* 20.6–33.5%).

**Conclusion:**

Our results indicated that HLJDD regulated blood circulation by inhibiting platelet aggregation and thrombosis, which might also extensively contribute to the clinical prevention and treatment of cerebrovascular diseases.

## Introduction

Antithrombosis is a critical treatment with anticoagulants to prevent stroke prevention, deep-vein thrombosis, heart attack, and pulmonary embolism. Many ingredients in herbal medicines have antithrombotic activities, such as afzeloside extracted from *Malus halliana* Koehne (Rosaceae) flowers (Cui et al. 2018), myricitrin isolated from *Cercis chinensis* Bunge (Leguminosae) leaves (He et al. 2019), compounds from *Radix Paeoniae* Rubra (Ranunculaceae) (Xie et al. 2017), and the procoagulant effects of constituents from *Cordyceps militaris* L. ex Fr. (Clavicipitaceae) link (Zhang et al. 2018).

As a famous prescription in China, Huanglian Jiedu Decoction (HLJJD) was found to perform neuroprotective effects with its major active compounds (containing 42.12% baicalin and 31.17% berberine), and possess cardioprotective effects *via* preventing pathological cardiac hypertrophy and regulating lipid metabolism (Fang et al. 2017; Chen et al. 2021). As an ancient prescription originated during the Tang Dynasty in China (618-906 A.D.), HLJJD consists of four Chinese medicines: the roots of *Coptis chinensis* Franch. (Ranunculaceae), roots of *Scutellaria baicalensis* Georgi. (Lamiaceae), dry bark of *Phellodendron chinense* C.K. Schneid. (Rutaceae), and ripe fruits of *Gardenia jasminoides* J. Ellis (Rubiaceae). This prescription was applied extensively in clinics for the treatment of cerebrovascular diseases, transient cerebral ischaemia, and Alzheimer's disease in China and Japan (Zhao et al. 2014; Gu et al. 2018; Chen et al. 2021). However, its antithrombotic effects against thrombosis and platelet aggregation were uncertain, which might contribute to the effect of HLJDD in treating stroke sequelae.

To study the mechanism of anti-ischaemic cerebrovascular disease in the aspect of the antithrombotic effect, we conducted a series of thrombotic experiments to study the antiplatelet aggregation of HLJDD and its potential active ingredients.

## Materials and methods

### Animals and chemical agents

Healthy male Wistar rats (8–10 weeks old, bodyweight 220 ± 25 g) were obtained from the Experimental Animal Centre of Jiangxi University of Traditional Chinese Medicine (License No.: SCXK2015-0378). The rats were fed with rodent food and water in a temperature- and humidity-controlled environment on a 12 h light/dark cycle, for at least 3 days before the experiments. The animal protocols were approved by the Ethics Committee of Jiangxi University of Science and Technology and followed the regulatory animal care guidelines of the United States National Institute of Health (Bethesda, MD, USA).

HLJDD contained 300 g roots of *Coptis chinensis*, 200 g roots of *Scutellaria baicalensis*, 200 g dry bark of *Phellodendron chinense*, and 300 g ripe fruits of *Gardenia jasminoides*. The water-soluble extract was obtained by two reflux extractions with the 10-times volume of water; the yield was 2.6%. The powder of the whole prescription was prepared by concentration under reduced pressure and spray drying.

Aspirin (Yangzhou Second Pharmaceutical Factory, Jiangsu, China); arachidonic acid (AA), ADP and collagen (Aldrich-Sigma, St. Louis, MO, USA); geniposide (≥95%, Sichuan Shuangziye Bio Sci Co., Ltd., Chengdu, China); baicalin and berberine (≥90%, Nanjing Zelang Medical Technology Co. Ltd., Nanjing, China). Prothrombin time (PT) reagents (Thermo Scientific Co. Ltd., Waltham, MA, USA); the chemicals or organic reagents for HPLC were of chromatographically pure grade.

### Rats MCAO modelling and antithrombotic studies

Adult rats were subjected to MCAO surgery according to the modified Longa suture method (Wang et al. 2019). After cerebral vascular blockage for 90 min, the filament was withdrawn to achieve reperfusion. The wound was treated with penicillin powder against microbial infection. Saline or HLJDD were treated by gavage for 3 days. The last administration was exerted at 60 min before antithrombotic studies. In addition, geniposide, berberine, and baicalin were treated by intravenous injection with last administration at 30 min before subsequent experiments. For the normal group, normal rats were subjected to antithrombotic studies directly (*n* = 4).

Twenty-four hours after MCAO surgery for the model control group and treatment subgroups (*n* = 4), arteriovenous shunt (silk thread model) was performed according to the previous report (Lorrain et al. 2003). Briefly, after anaesthetization with pentobarbital sodium (i.p.), the right carotid artery and the left jugular vein of rats were inserted separately with 10 cm long polyethylene tubes (1 mm i.d., linked by a central part (8 cm long; 2 mm i.d.) containing a 6 cm silk thread in 200 U/mL heparin). This central part of the shunt was removed after 15 min of blood circulation and the silk thread with thrombus attached was collected and weighted after dried at 80 °C for 1 h.

Twenty-four hours after MCAO surgery for the model control group and treatment subgroups (*n* = 4), orbital blood was collected in a citrated tube. PT was determined using a Thrombotimer (Behnk Elektronik, Germany) according to the previous description (Kim et al. 2012). Citrated rat plasma (100 µL) was preincubated with 200 μL of PT assay reagent for 10 min at 37 °C, and clotting time was recorded. PT results were expressed in seconds. Each sample was tested in triplicate.

This test was performed in treatment subgroups of HLJDD and its active ingredients. Twenty-four hours after MCAO surgery for the model control group and treatment subgroups (*n* = 4), platelet-rich plasma (PRP) was prepared by centrifugation of citrated rat plasma at 1000 rpm for 5 min and further centrifuged at 3000 rpm for 10 min to prepare platelet-poor plasma. Platelet aggregation was measured by a turbidimetric method using a whole-blood aggregometer (WBA analyzer; Mebanix, Tokyo, Japan). Briefly, 200 µL of rat PRP was incubated at 37 °C and stirred at 1000 rpm for 3 min in the aggregometer. Subsequently, 2 µg/mL of collagen, 1.0 mM arachidonic acid, or 1.0 μM ADP was added to stimulate platelet aggregation in phosphate buffer solution (PBS). Changes in light transmission were tested for 5 min and the maximal aggregation rate was recorded (Day et al. 2006).

The adhesion of blood platelet to collagen type I was determined with Tuszynski’s and Murphy’s method (Olas et al. 2017). After a 96-well microtiter dish incubated with 50 µL of fibrinogen for 2 h and washed with PBS, the wells were supplemented with 50 µL of 10 µM ADP. Then 100 µL of platelet suspension was added to each well and the plate was incubated at 37 °C for 1 h. Non-adherent cells were removed by aspiration and the wells were washed with PBS three times. The total cell-associated protein was determined by dissolving the attached blood platelets directly in the microtiter wells with 200 µL of working solution of bicinchoninic acid protein assay kit (Thermo Scientific, Rockford, IL, USA), and incubated at 37 °C for 60 min. The absorbance of each well was determined at 560 nm with a microtiter plate reader (Thermo Labsystems, Vantaa, Finland).

### The main components of HLJDD in HPLC test

The platform of Agilent 1100 High-Performance Liquid Chromatograph (Beijing Keyi Hengda Technology Co., Ltd., Beijing, China) was applied to study the main components of HLJDD. Chromatographic column: Agilent TC-C_18_ (4.6 × 250 mm, 5 μm); mobile phase: acetonitrile (A)-0.2% phosphoric acid water (B); linear-gradient elution program: 0–10 min, 10–22% A; 10–40 min, 22–23% A; 40–45 min, 23–24% A; 45–60 min, 24% A; detection wavelength: 260 nm; flow rate: 1.0 mL/min; sample volume: 10 μL; column temperature: 25 °C. The sample for determination was prepared by resolving 10 mg powder in 5 mL of 30% methanol and filtered with 0.45 μm microporous membrane.

### Statistical analysis

All data were expressed in the form of mean ± SEM. Data were analyzed by one-way ANOVA, followed by Student’s two-tailed *t*-test for comparison between two groups. *p* < 0.05 was considered as a significant difference between each group.

## Results

### Antithrombotic activity of HLJDD in MCAO rats

**Figure 1.**
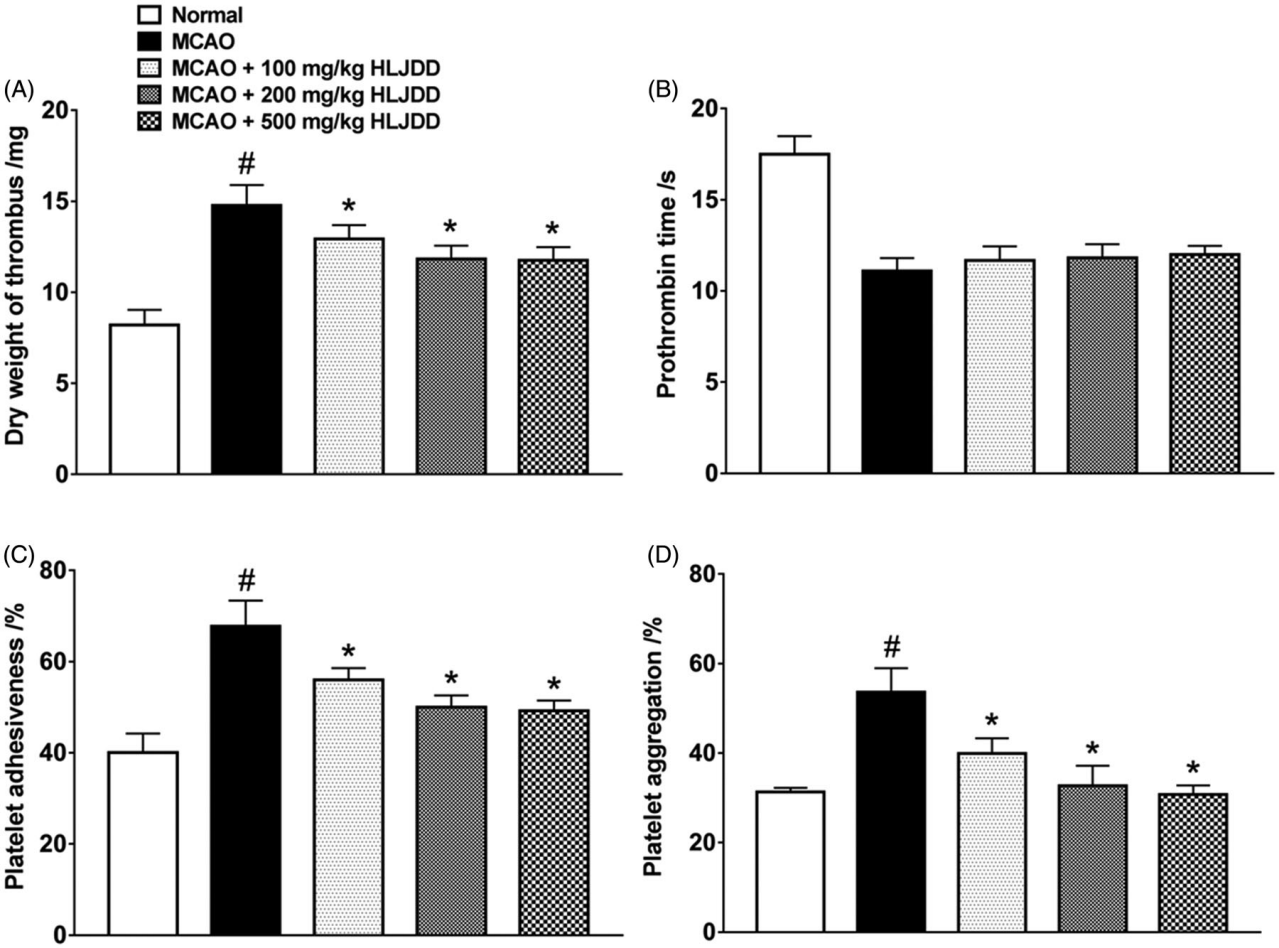
Antithrombotic effects of HLJDD in MCAO rats. (A) The dry weight of thrombus in arteriovenous shunt model at 24 h after MCAO surgery. (B) PT was determined in orbital blood at 24 h after MCAO onset. (C) The adhesion of blood platelet to collagen type I was determined with Tuszynski’s and Murphy’s method at 24 h after MCAO onset. (D) Platelet aggregation was measured by a turbidimetric method using a whole-blood aggregometer. The data expression method is mean ± SEM (*n* = 4). # denotes statistical significance (*p* < 0.05) compared with the normal group; *p < 0.05, significantly different from the model group by one-way ANOVA followed by *post-hoc* Student–Newman–Keuls test or unpaired and two-tailed Student's *t*-test.

Many indicators were studied to evaluate the antithrombotic activity of HLJJD in MCAO rats, such as thrombosis, PT, adhesion of blood platelet, and platelet aggregation. HLJJD did not influence the change of PT, but significantly inhibited thrombosis by 12.8–20.6% in reducing the dry weight of thrombus, and blocked collagen-induced platelet aggregation by 25.6–42.7% and adhesion of blood platelet by 17.3–27.3% in a dose-dependent manner (Figures 1(A–D)). The IC_50_ value of HLJJD on antiplatelet aggregation was 670 mg/kg.

### HPLC assay of main components in HLJDD

**Figure 2.**
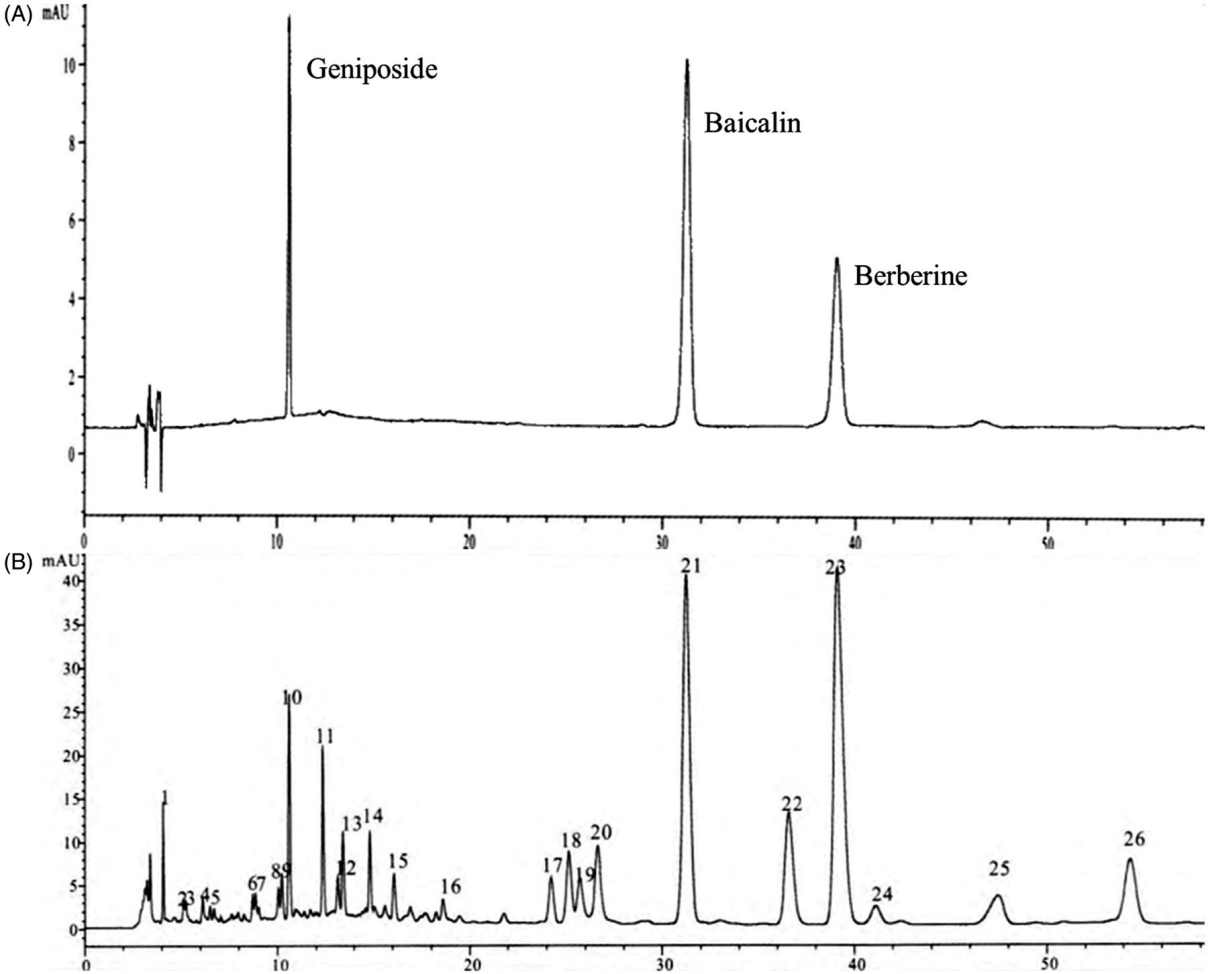
HPLC analysis of HLJDD as the powder of the whole prescription. (A) Representative peaks of standard chemicals as the main ingredients of HLJDD. (B) HPLC chart of HLJDD was measured by DAD multi-wavelength detection at the wavelength of 260 nm.

High-performance liquid chromatography was applied to analyze the main ingredients in HLJDD. Due to the complexity of components, the selection of detection wavelengths greatly impacted the confirmation of different compounds. It was confirmed that the peaks of the main components geniposide, baicalin, and berberine could be completely separated when detected at the wavelength of 260 nm, with high detection sensitivity and large peaks (Figures 2(A,B)).

### Antithrombotic effects of main ingredients on platelet aggregation

**Figure 3.**
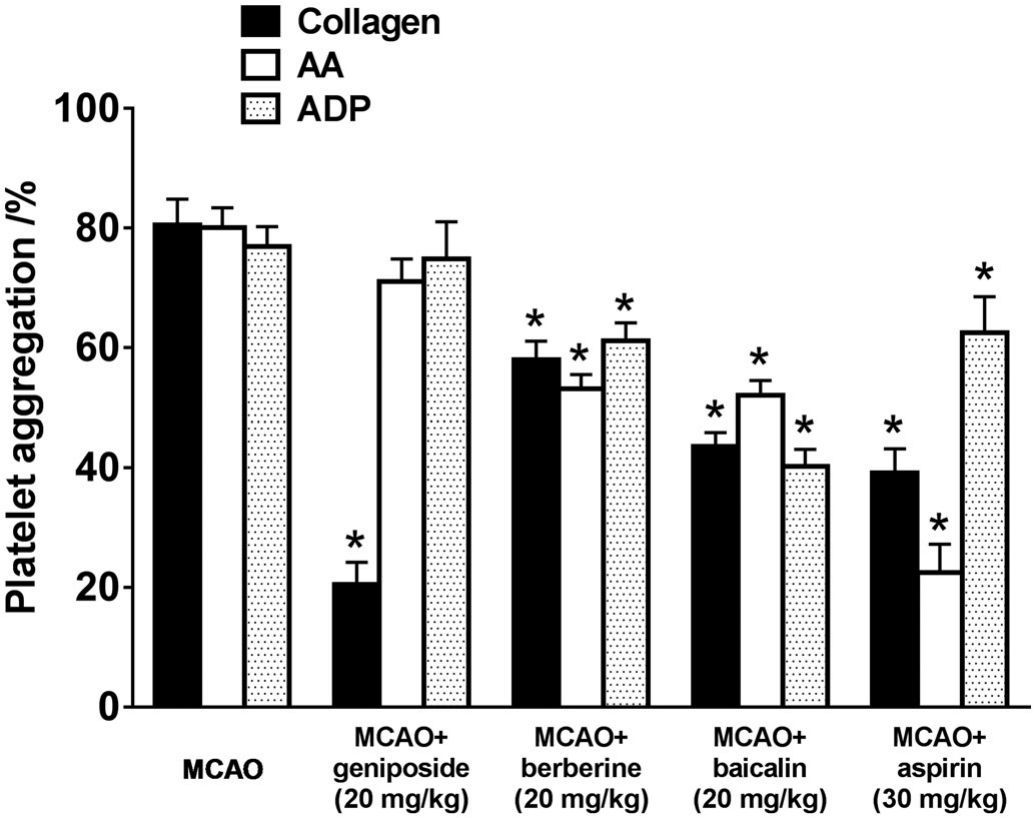
Antiplatelet aggregation of main ingredients of HLJDD in MCAO rats. Platelet aggregation was measured by a turbidimetric method using a whole-blood aggregometer. The data expression method is mean ± SEM (*n* = 4). *p < 0.05, significantly different from the model group by one-way ANOVA followed by *post-hoc* Student–Newman–Keuls test or unpaired and two-tailed Student's *t*-test.

The antithrombotic effects of geniposide, baicalin, and berberine were tested on platelet aggregation induced by AA, ADP, and collagen, respectively (Figure 3). Our results indicated that geniposide only performed antiplatelet aggregation induced by collagen, but not AA or ADP. Both baicalin and berberine showed markedly antiplatelet aggregation induced by all activators. The antithrombotic activity of baicalin was relatively higher than that of berberine (35.0–47.8% *vs.* 20.6–33.5%).

## Discussion and conclusions

Ischaemic stroke can induce the blood to be in a state of high concentration, high viscosity, and high degree of aggregation (Grau et al. 1994, Hovhannesyan and Hovhannisyan 2019). In our study, the weight of thrombus increased significantly at 24 h after MCAO onset. HLJDD could significantly inhibit thrombosis by improving platelet adhesion and platelet aggregation but had no effect on the clotting time of PT. It was assumed that the active ingredients in HLJDD might play an antithrombotic effect mainly by inhibiting the activation of platelet function. A review of the literature revealed the antithrombotic activities of many other decoctions were reported, such as Sheng Hua decoction, Buyang Huangwu decoction, Yiqi Huoxue decoction, etc. (Qian and Yu 2011; Liao et al. 2018; Wu et al. 2019). Their activities were relatively moderate with antiplatelet aggregation of 20–40%, similar to HLJDD.

In subsequent antiplatelet experiments, it was found that at 24 h after MCAO surgery, platelet adhesion increased obviously and the rates of platelet aggregation induced by ADP, collagen, and AA were significantly enhanced. Geniposide exhibited the strongest inhibitory effect on platelet aggregation induced by collagen with high specificity. The collagen-induced platelet aggregation is closely related to the release and metabolism of AA, which suggests that the anti-platelet aggregation of geniposide may be related to its adjustment to the metabolism of AA. Our result on geniposide is consistent with previous related research (Suzuki et al. 2001; Zhang et al. 2013). Baicalin was studied only on ADP-induced platelet aggregation in a former study (Lee et al. 2015). In addition, there was no related report on the anticoagulant activity of berberine.

In conclusion, the extract of HLJDD possessed the effect of promoting blood circulation, which was consistent with antiplatelet aggregation of baicalin and berberine, as the main components of the prescription. The activation of HLJDD on blood circulation will become one of its important pharmacological mechanisms for the clinical prevention and treatment of cerebrovascular diseases.

## Data Availability

Additional information and requests for data should be directed to the corresponding author X. Y. Chen (chenxyan5@126.com).
